# Supplementary figures and images for: Ambient Air Pollution and Risk of Admission Due to Asthma in the Three Largest Urban Agglomerations in Poland: A Time-Stratified, Case-Crossover Study
Source: Int J Environ Res Public Health. 2022 May 14;19(10):5988. doi: 10.3390/ijerph19105988 (PMC9140383; doi:10.3390/ijerph19105988)

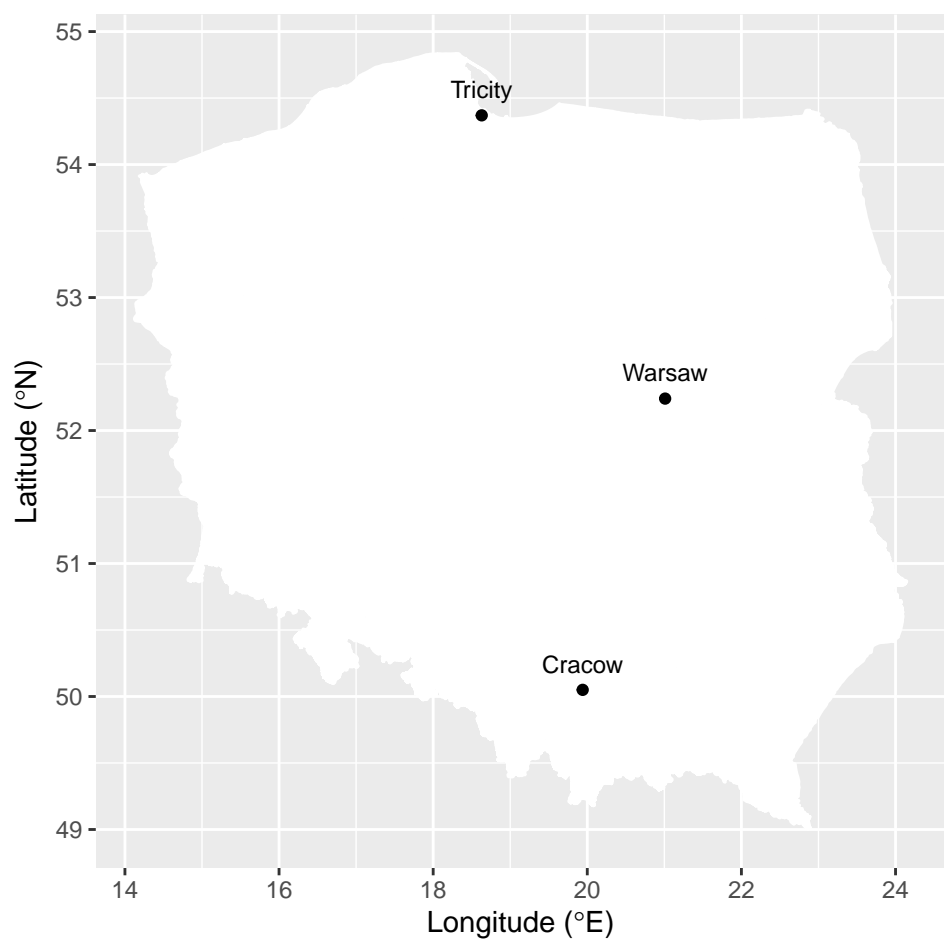

Supplement: Supplementary file 1 [file ijerph-19-05988-s001.zip › Fig S1.pdf]
